# Supplementary material for: Associations of ultrasound-based inflammation patterns with peripheral innate lymphoid cell populations, serum cytokines/chemokines, and treatment response to methotrexate in rheumatoid arthritis and spondyloarthritis
Source: PLoS One. 2021 May 21;16(5):e0252116. doi: 10.1371/journal.pone.0252116 (PMC8139502; doi:10.1371/journal.pone.0252116)
Supplement: S1 Table — (DOCX) [file pone.0252116.s003.docx]

**S1 Table. Differences in serum cytokine/chemokine levels between rheumatoid arthritis and spondyloarthritis**

|  | Rheumatoid arthritis  (n=65) | Spondyloarthritis  (n=34) | p-value |
| --- | --- | --- | --- |
| β-defensin2, ng/ml | 0.209 (0.094-0.397) | 1.77 (0.595-5.06) | <0.0001 |
| Calprotectin, μg/ml | 2.94 (2.35-3.75) | 2.69 (2.22-3.75) | 0.5012 |
| CCL20/MIP3a, pg/ml | 31.4 (15.9-64.8) | 13.3 (1.27-29.8) | 0.0012 |
| C-reactive protein, mg/dl | 0.270 (0.075-1.92) | 0.340 (0.09-1.47) | 0.8968 |
| GM-CSF, pg/ml | 12.4 (0.00-96.5) | 0.00 (0.00-7.38) | 0.0005 |
| IFN-γ, pg/ml | 4.78 (1.50-18.5) | 1.21 (0.00-3.90) | <0.0001 |
| IL-1β, pg/ml | 1.51 (0.00-4.79) | 0.138 (0.00-1.08) | 0.0038 |
| IL-6, pg/ml | 3.70 (0.00-20.3) | 0.00 (0.00-4.38) | 0.0426 |
| IL-8, pg/ml | 13.2 (6.82-41.5) | 16.5 (7.92-60.2) | 0.5141 |
| IL-9, pg/ml | 0.00 (0.00-17.4) | 0.00 (0.00-0.464) | 0.0762 |
| IL-10, pg/ml | 0.118 (0.00-1.94) | 0.00 (0.00-0.87) | 0.3849 |
| IL-12p70, pg/ml | 2.89 (1.21-12.1) | 2.70 (1.24-3.83) | 0.1687 |
| IL-15, pg/ml | 1.96 (0.00-10.4) | 0.00 (0.00-2.46) | 0.0026 |
| IL-17A, pg/ml | 1.07 (0.00-4.58) | 0.0231 (0.00-2.06) | 0.0324 |
| IL-17F, pg/ml | 3.93 (0.00-133.3) | 0.00 (0.00-1.87) | 0.0004 |
| IL-21, pg/ml | 10.8 (5.15-27.0) | 12.4 (6.99-16.9) | 0.7957 |
| IL-22, ng/ml | 0.00 (0.00-2.51) | 0.00 (0.00-0.00) | <0.0001 |
| IL-23, ng/ml | 0.480 (0.00-7.67) | 0.00 (0.00-0.012) | <0.0001 |
| Lipocalin-2/NGAL, μg/ml | 0.365 (0.204-0.658) | 0.438 (0.321-0.683) | 0.0527 |
| TNF-α, pg/ml | 11.0 (7.39-20.9) | 12.0 (6.54-25.8) | 0.8702 |

Shown are medians with interquartile ranges in parenthesis.
