# Supplementary material for: Associations of ultrasound-based inflammation patterns with peripheral innate lymphoid cell populations, serum cytokines/chemokines, and treatment response to methotrexate in rheumatoid arthritis and spondyloarthritis
Source: PLoS One. 2021 May 21;16(5):e0252116. doi: 10.1371/journal.pone.0252116 (PMC8139502; doi:10.1371/journal.pone.0252116)
Supplement: S2 Table — (DOCX) [file pone.0252116.s004.docx]

**S2 Table. Differences in serum cytokine/chemokine levels between groups based on ultrasound**

|  | Group 1  (Synovitis-dominant)  (n=37) | Group 2  (Synovitis-nondominant)  (n=62) | p-value |
| --- | --- | --- | --- |
| β-defensin2, ng/ml | 0.232 (0.095-0.478) | 0.394 (0.139-1.75) | 0.0256 |
| Calprotectin, μg/ml | 3.51 (2.49-4.06) | 2.62 (1.93-3.26) | 0.0020 |
| CCL20/MIP3a, pg/ml | 34.4 (17.2-77.5) | 21.2 (6.02-40.5) | 0.0282 |
| C-reactive protein, mg/dl | 0.970 (0.365-3.26) | 0.135 (0.050-0.733) | <0.0001 |
| GM-CSF, pg/ml | 0.00 (0.00-90.2) | 0.00 (0.00-37.0) | 0.6965 |
| IFN-γ, pg/ml | 2.79 (1.29-16.0) | 3.66 (0.440-7.80) | 0.7428 |
| IL-1β, pg/ml | 0.637 (0.00-3.10) | 0.483 (0.00-2.78) | 0.7344 |
| IL-6, pg/ml | 4.62 (0.00-22.0) | 0.00 (0.00-13.6) | 0.1413 |
| IL-8, pg/ml | 15.6 (8.47-31.0) | 13.3 (6.41-54.2) | 0.9250 |
| IL-9, pg/ml | 0.00 (0.00-19.7) | 0.00 (0.00-12.6) | 0.6623 |
| IL-10, pg/ml | 0.536 (0.00-1.94) | 0.00 (0.00-0.87) | 0.2274 |
| IL-12p70, pg/ml | 2.44 (0.986-13.3) | 2.70 (1.53-5.30) | 0.7686 |
| IL-15, pg/ml | 1.97 (0.00-8.90) | 0.054 (0.00-5.96) | 0.3852 |
| IL-17A, pg/ml | 0.38 (0.00-2.33) | 1.19 (0.00-4.13) | 0.4876 |
| IL-17F, pg/ml | 0.00 (0.00-123) | 0.00 (0.00-35.5) | 0.6722 |
| IL-21, pg/ml | 8.70 (5.41-26.9) | 13.1 (6.99-21.2) | 0.5823 |
| IL-22, ng/ml | 0.00 (0.00-2.08) | 0.00 (0.00-0.477) | 0.6352 |
| IL-23, ng/ml | 0.00 (0.00-5.27) | 0.00 (0.00-2.23) | 0.5646 |
| Lipocalin-2/NGAL, μg/ml | 0.296 (0.205-0.619) | 0.424 (0.253-0.729) | 0.1702 |
| TNF-α, pg/ml | 11.7 (6.90-26.0) | 11.3 (7.43-22.3) | 0.8795 |

Shown are medians with interquartile ranges in parenthesis.
