# Supplementary material for: Associations of ultrasound-based inflammation patterns with peripheral innate lymphoid cell populations, serum cytokines/chemokines, and treatment response to methotrexate in rheumatoid arthritis and spondyloarthritis
Source: PLoS One. 2021 May 21;16(5):e0252116. doi: 10.1371/journal.pone.0252116 (PMC8139502; doi:10.1371/journal.pone.0252116)
Supplement: S3 Table — (DOCX) [file pone.0252116.s005.docx]

**S3 Table. Correlations between serum cytokine/chemokine levels and numbers of peripheral innate lymphoid cell populations**

|  | ILC1 | ILC2 | ILC3 |
| --- | --- | --- | --- |
| β-defensin2 | 0.0739 | 0.0676 | 0.1790 |
| Calprotectin | –0.1747 | –0.1060 | –0.1057 |
| CCL20/MIP3a | –0.0239 | –0.0153 | 0.0373 |
| C-reactive protein | –0.1175 | –0.1267 | –0.1655 |
| GM-CSF | 0.1077 | 0.0164 | 0.1662 |
| IFN-γ | –0.0030 | –0.0344 | 0.0449 |
| IL-1β | –0.0516 | –0.0404 | 0.0462 |
| IL-6 | –0.0996 | –0.1108 | –0.0825 |
| IL-8 | –0.0670 | 0.0500 | 0.0843 |
| IL-9 | 0.0641 | 0.1514 | 0.1851 |
| IL-10 | –0.2756** | –0.1942 | –0.1775 |
| IL-12p70 | –0.0199 | –0.0667 | 0.0844 |
| IL-15 | –0.1102 | –0.0599 | –0.0334 |
| IL-17A | 0.1178 | 0.0070 | 0.1305 |
| IL-17F | –0.0756 | –0.0410 | 0.0393 |
| IL-21 | –0.1221 | –0.0991 | 0.0170 |
| IL-22 | –0.1492 | –0.0605 | –0.0357 |
| IL-23 | –0.1882 | –0.1491 | –0.0961 |
| TNF-α | –0.1404 | –0.0613 | –0.0076 |
| Lipocalin-2/NGAL | 0.0129 | 0.0993 | 0.2135* |

Spearman’s correlation coefficients are presented. * p-value<0.05. ** p-value<0.01.

ILC1: group 1 innate lymphoid cell; ILC2: group 2 innate lymphoid cell; ILC3: group 3 innate lymphoid cell
