# Supplementary material for: Associations of ultrasound-based inflammation patterns with peripheral innate lymphoid cell populations, serum cytokines/chemokines, and treatment response to methotrexate in rheumatoid arthritis and spondyloarthritis
Source: PLoS One. 2021 May 21;16(5):e0252116. doi: 10.1371/journal.pone.0252116 (PMC8139502; doi:10.1371/journal.pone.0252116)
Supplement: Attachment — Submitted filename: Response.docx [file pone.0252116.s006.docx]

Reviewer #1

We thank the Reviewer #1 for kind comments and suggestions.

**> My primary concern regarding the study is if there was only one person performing ultrasound exams or were there more examiners? Also, was there examiner blinding regarding the clinical diagnosis or not? Noting this in methodology section and entering short discussion about possible (dis)advantages of approach used in the study is crucial for better interpretation of the results. If this is added to the manuscript, I would firmly approve it for publishing.**

We appreciate the Reviewer pointing this out. We initially included a sentence for these pieces of information in the manuscript, which was removed later during our editing process for some reason. In our study, multiple examiners performed ultrasound exams. They were instructed not to see the patient’s clinical information including the diagnosis, but there was no strict restriction defined by the protocol that prevented them from accessing the patient’s clinical information. We completely agree that these are important pieces of information. We have added them and discussed the advantages/disadvantages.

Page 7, 2^nd^ paragraph:

“Ultrasound was performed by either one of the 10 trained sonographers (MK, KIkeda, TS, KIida, KSuga, NN, NM, TKasuya, TKumagai, SF) who were instructed not to see the patient’s clinical information.”

Page 21, 3^rd^ paragraph:

“Third, ultrasound was performed by multiple examiners. This probably has increased the heterogeneity of evaluation, while the heterogeneity can make the results more generalizable. Fourth, these sonographers were not completely blinded to the clinical information (i.e., they could have access if they intended to), which could have biased their evaluation.”

We thank again the Reviewer #1 for taking time to review our manuscript.

Reviewer #2

We thank the Reviewer #2 for pointing out our mistake.

**> In the Materials and methods you explain that you performed ultrasound examination for enthesitis at extensor digitorum insertions into 1st-5th middle phalanx, while in Figure 1 extensor digitorum insertion into proximal phalanx is shown.
Which site is correct? Could you name reference for evaluating this enthesis site?**

The Fig.1 legend is correct. We corrected the description in the Materials and methods section. To our knowledge, there is no report that we can cite on the ultrasound assessment of the enthesis at this part.

Page 8, 1^st^ paragraph:

“For enthesitis, we scanned 12 entheseal sites bilaterally: extensor digitorum insertions into 1^st^-5^th^ proximal phalanx,”

We thank again the Reviewer #2 for taking time to review our manuscript.

Academic Editor

We thank the Academic Editor for helpful suggestions.

**> 1. Please ensure that your manuscript meets PLOS ONE's style requirements, including those for file naming. The PLOS ONE style templates can be found at**

[**https://journals.plos.org/plosone/s/file?id=wjVg/PLOSOne_formatting_sample_main_body.pdf**](https://journals.plos.org/plosone/s/file?id=wjVg/PLOSOne_formatting_sample_main_body.pdf) **and**

[**https://journals.plos.org/plosone/s/file?id=ba62/PLOSOne_formatting_sample_title_authors_affiliations.pdf**](about:blank)

We have corrected the file names for Figures. We reviewed the samples and instructions and think that our manuscript now meets PLOS ONE’s style requirements. However, we are happy to revise the manuscript if there is any other thing we missed.

**> 2. In your Methods section, please provide additional information about the participant recruitment method and the demographic details of your participants. Please ensure you have provided sufficient details to replicate the analyses such as descriptions of where participants were recruited and where the research took place.**

We have modified the description for participant recruitment method. Demographic information is provided in Table 1.

Page 6, 1^st^ paragraph:

“We screened consecutive patients with either RA or SpA including PsA who had peripheral symptoms upon a visit at the outpatient clinic of Department of Allergy and Clinical Immunology between August 2018 and February 2020.”

“We enrolled patients when they gave written informed consent to participate in the study, and they did not receive any study procedures before giving it.”

**> 3. Please provide a sample size and power calculation in the Methods, or discuss the reasons for not performing one before study initiation.**

We did not perform sample-size/power calculation. We have mentioned this and provided the reason in the revised manuscript.

Page 10, 1^st^ paragraph:

“Given the exploratory nature of this study, we did not perform sample size or power calculation.”

**> 4. Please note that PLOS does not permit references to 'data not shown.' Authors should provide the relevant data within the manuscript, the Supporting Information files, or in a public repository. If the data are not a core part of the research study being presented, we ask that authors remove any references to these data.**

We have removed all “data not shown” references from our revised manuscript.

We thank again the Academic Editor for taking time to review our manuscript.
